# The impact of supply-side and demand-side interventions on use of antenatal and maternal services in western Kenya: a qualitative study

**DOI:** 10.1186/s12884-020-03130-4

**Published:** 2020-08-08

**Authors:** Mitsuaki Hirai, Jamae Morris, Jill Luoto, Rosebel Ouda, Nancy Atieno, Robert Quick

**Affiliations:** 1 Division of Global Health Protection, Office of the Director, Center for Global Health, Centers for Disease Control and Prevention, 1600 Clifton Road NE, Atlanta, GA 30329 USA; 2 Department of African American Studies, Georgia State University, 33 Gilmer Street SE, Atlanta, GA 30303 USA; 3 RAND Corporation, Santa Monica, CA 90407 USA; 4 Safe Water and AIDS Project, P.O Box 3323, Kisumu, 40100 Kenya; 5 National Center for Emerging and Zoonotic Infectious Diseases, Office of Infectious Diseases Centers for Disease Control and Prevention, 1600 Clifton Road NE, Atlanta, GA 30329 USA

**Keywords:** Maternal and child health, Qualitative research, Antenatal care, Kenya

## Abstract

**Background:**

Antenatal care (ANC) and delivery by skilled providers have been well recognized as effective strategies to prevent maternal and neonatal mortality. ANC and delivery services at health facilities, however, have been underutilized in Kenya. One potential strategy to increase the demand for ANC services is to provide health interventions as incentives for pregnant women. In 2013, an integrated ANC program was implemented in western Kenya to promote ANC visits by addressing both supply- and demand-side factors. Supply-side interventions included nurse training and supplies for obstetric emergencies and neonatal resuscitation. Demand-side interventions included SMS text messages with appointment reminders and educational contents, group education sessions, and vouchers to purchase health products.

**Methods:**

To explore pregnant mothers’ experiences with the intervention, ANC visits, and delivery, we conducted focus group discussions (FGDs) at pre- and post-intervention. A total of 19 FGDs were held with pregnant mothers, nurses, and community health workers (CHWs) during the two assessment periods. We performed thematic analyses to highlight study participants’ perceptions and experiences.

**Results:**

FGD data revealed that pregnant women perceived the risks of home-based delivery, recognized the benefits of facility-based delivery, and were motivated by the incentives to seek care despite barriers to care that included poverty, lack of transport, and poor treatment by nurses. Nurses also perceived the value of incentives to attract women to care but described obstacles to providing health care such as overwork, low pay, inadequate supplies and equipment, and insufficient staff. CHWs identified the utility and limitations of text messages for health education.

**Conclusions:**

Future interventions should ensure that adequate workforce, training, and supplies are in place to respond to increased demand for maternal and child health services stimulated by incentive programs.

## Background

High maternal and neonatal mortality remain key public health issues in Kenya. In 2015, the maternal mortality ratio was estimated to be 510 per 100,000 live births, which was higher than that of neighboring countries, including Uganda (343/100,000), Ethiopia (353/100,000) and Tanzania (398/100,000) [1]. In the same year, over 33,000 newborns lost their lives in Kenya during the neonatal period, within 28 days of birth [2].

Antenatal care (ANC) and delivery with skilled birth attendants are known to be effective strategies to improve clinical outcomes [3] and prevent maternal and neonatal mortality [4]. Based on empirical evidence [5], the World Health Organization (WHO) has promoted the focused antenatal care (FANC) model in developing countries where pregnant mothers were encouraged to visit ANC facilities at least four times prior to delivery [6].

ANC and delivery services at health facilities have been relatively underutilized in Kenya [2]. In 2014, only 58% of women visited ANC clinics four times or more during their pregnancy, and 37% of them did not deliver at health facilities [2]. Moreover, 43% of mothers had no postnatal checkup [2].

Under-utilization of ANC services can be influenced by a number of supply and demand-side factors. Supply-side determinants include the availability of healthcare providers, staff training, and medical supplies [7–11]. Demand-side determinants include cost, household wealth levels, maternal education, previous experience of obstetric complications, previous experiences at health care facilities, physical access to healthcare facilities, infectious disease testing, and cultural beliefs on pregnancy [9–16]. In western Kenya, monitoring fetal position, offering vaccinations, and providing medications were found to be major facilitators for ANC visits while clinic staff’s negative attitudes and behaviors, long waiting times for services, and cost were identified as barriers [13]. Human immunodeficiency virus (HIV) testing has been identified as both a facilitator and a barrier to obtaining care [13].

One potential strategy to increase the demand for ANC services is to provide effective and desirable health interventions as incentives to motivate service use. In Malawi, the Ministry of Health, the United Nations Children’s Fund (UNICEF), and Population Services International (PSI) implemented an antenatal care program in which pregnant women received free water, sanitation, and hygiene (WASH) products (e.g., soap, water treatment supplies) up to four times during pregnancy to incentivize ANC service use [17]. This program contributed to an increase in the percentage of women who visited antenatal care clinics at least four times and delivered in healthcare facilities. A similar program in Kenya also increased ANC service utilization, facility-based delivery, and postnatal checkups among pregnant women [14].

### ANC program in Western Kenya

From November 2013 to October 2014, the Safe Water and AIDS Project (SWAP), a Kenyan non-governmental organization (swapkenya.org), implemented an integrated ANC program in western Kenya in collaboration with the local offices of the Kenyan Ministry of Health, the Rand Corporation, and Centers for Disease Control and Prevention (CDC) to promote ANC visits by addressing both supply- and demand-side factors. Supply-side interventions consisted of two components to improve quality of care: 1) short trainings for nurses and healthcare providers on management of obstetric emergencies, neonatal resuscitation, patient-centered care; and 2) provision of medical and health facility supplies including handwashing stations, blood pressure cuffs, and bulb suction devices and ambu-bags for neonatal resuscitation.

Demand-side interventions also included three approaches: 1) as an incentive for women to attend ANC clinics and deliver at healthcare facilities, pregnant women enrolled in the study received free vouchers (100 Kenyan Shillings or approximately 1 USD) to purchase SWAP products, such as soap and water treatment supplies; 2) pregnant women with the same expected month of delivery were enrolled into cohorts for group education sessions during which nurses discussed a wide range of health topics (e.g., malaria prevention, breastfeeding, water treatment, handwashing, and nutrition) and community health workers (CHWs) facilitated group discussions about the experience of pregnancy; and 3) participants received health education messages and ANC appointment reminders by short message service (SMS) text messages.

After the SWAP intervention infrastructure had been set up and as baseline data were being collected, we discovered that the Government of Kenya, in collaboration with a German bank, had initiated a program called Output-Based Aid (OBA) in intervention healthcare facilities in September 2013. This program, which we had not anticipated, was targeted toward poor and underserved groups, and enabled women to receive ANC, free deliveries at the health care facility of their choice (including private facilities), postnatal care, and emergency care free of charge after paying a registration fee of 100 Kenya shillings (about 1 USD).

CDC and Rand, with the assistance of SWAP’s research team, conducted a controlled quantitative evaluation of the SWAP intervention in 20 public healthcare facilities, comparing maternal registry data from 10 healthcare facilities in two intervention subcounties to data from 10 healthcare facilities in two control subcounties (geographically remote from intervention subcounties) during a baseline period from January through September 2013 and a follow-up (post-intervention) period from January through September 2014 (Harvey R, personal communication). Because of the unanticipated OBA intervention, which enabled mothers to obtain free care from either public or private healthcare facilities, we expanded quantitative data collection to include maternal registry data from the 7 private healthcare facilities in the intervention subcounties and the 5 private healthcare facilities in the comparison subcounties.

Results of the quantitative evaluation suggested that, from baseline to follow-up, the change in attendance at 4 or more antenatal clinic visits from baseline to follow-up among mothers was 1.6 times greater in public intervention than public control healthcare facilities, and 1.7 times greater in private intervention than private control healthcare facilities. The change in healthcare facility deliveries from baseline to follow-up was 1.2 times higher in public intervention than public control healthcare facilities and 2.2 times higher in private intervention than private control healthcare facilities. Although results were confounded by the simultaneous implementation of two interventions in the same district, increased use of private healthcare facilities, which were not included in the SWAP intervention, suggests that the OBA intervention stimulated use of these higher-cost healthcare facilities, particularly for deliveries.

Qualitative research was incorporated into the study design to provide contextual background for quantitative findings. Focus group discussions (FGDs) were conducted before and after the interventions. Pre-intervention FGDs aimed to obtain baseline impressions of mothers and nurses on quality of care and barriers to healthcare services. Post-intervention FGDs explored how the opinions from baseline changed and how mothers, nurses, and community health workers perceived the impact of SWAP and OBA interventions on healthcare service utilization and healthcare workers’ ability to perform their jobs. All FGDs were held at SWAP intervention facilities. Data were not collected in the comparison healthcare facilities because mothers and nurses would have had no experience with the SWAP and OBA interventions.

## Methods

### Study sites

This qualitative research was conducted in the two intervention subcounties in the region in western Kenya formerly designated as Nyanza Province (we did not collect qualitative data in the comparison healthcare facilities because there were no interventions to discuss). Intervention healthcare facilities from which providers and expectant mothers were selected were located in Nyando and Nyakach subcounties in Kisumu County. Residents primarily speak DhoLuo and make a living through farming and fishing. Under-five mortality rates in in western Kenya (82 deaths per 1000 live births) are the highest in the country [2].

### Research team and reflexivity

Our research team consisted of public health scientists and SWAP staff. At the time of the study, the first author (MH) was a doctoral student at the George Washington University, the second (JM) and the last authors (RQ) were public health scientists at CDC, the third author was a senior scientist at RAND Corporation, and the rest of co-authors (RO and NA) were qualitative research assistants at SWAP from the Kisumu area who were fluent DhoLuo and English speakers. Both male and female researchers participated in this study. Before conducting this qualitative study, authors had gained adequate research expertise and experience; MH had been trained in mixed-methods research, anthropology, and global health, JM had specialized in qualitative methods and anthropological research, JL was trained as a behavioral economist and had extensive research experience in western Kenya, RQ had conducted many public health intervention studies in Kenya, and RO and NA had received qualitative research methods training and had established connections with local communities through SWAP.

### Study participants and data collection

Study participants were identified and recruited at intervention healthcare facilities through purposive sampling to specifically explore perspectives of pregnant women, nurses, and CHWs [18]. For the pre-intervention assessment, pregnant women who were 15 years old or older and nurses were recruited at SWAP intervention facilities (see Table 1) in person to participate in this study. The follow-up study recruited new mothers, nurses, and CHWs in SWAP intervention healthcare facilities listed in Table 1. Before both the pre- and post-intervention assessments, questions and prompts included in the FGDs were piloted with individuals not included in the study (Supplementary file 1).

**Table 1:** Timing, participant category, number of focus group discussions (FGDs), number of participants, and location of focus group discussions in western Kenya (2013–2014)

	Number of FGDs	Number of Participants (Range)	Facility Locations
Pre-intervention (2013)			
Mothers	4	6–8	Nyang’oma, Katito, Sondu, and Masogo
Nurses	4	7–10	Katito, Muhoroni, Masogo, and Sondu
Post-intervention (2014)			
Mothers	5	8–10	Muhoroni, Masogo, Nyang’oma, Katito, and Nyakach
Nurses	4	7–9	Muhoroni, Masogo, Katito, and Sondu
CHW	2	9–10	Awasi^a^, Katito

^a^ One post-intervention FGD was held at SWAP office in Awasi. All other FGDs were held at health facilities

Data were collected by trained facilitators who speak both English and DhoLuo. A total of eight FGDs (four for mothers, four for nurses) were held before the intervention was implemented, and 11 FGDs (five for mothers, four for nurses, two for CHWs) were conducted at follow-up. Each focus group consisted of six to 12 participants and lasted approximately 1.5 h. All FGDs took place at health facilities in locations where interruptions could be minimized. At the beginning of each FGD, a facilitator obtained oral consent from all study participants after explaining the purpose of this study, protection of confidentiality, and use of an audio-recording device for transcription purposes. A written consent was not obtained due to a concern of low literacy in study communities and to protect privacy of participants by having no written records with their personal identifiers and signatures. FGDs were recorded, transcribed verbatim in Luo and translated into English.

### Data analysis

This qualitative study conducted thematic analysis as the main analytical approach [18]. Two authors (MH and JM) read each transcript three to five times prior to data analysis. The authors who analyzed the data (MH and JM) did not moderate the FGDs or transcribe and translate FGD data. Data were analyzed with NVivo 11 through development of codes by constantly comparing them with the content of transcripts (i.e., open coding) and identifying a key code that represents several open coding categories and creating groups of data (i.e., axial coding). Through the open coding and axial coding, a total of five codebooks were developed by MH and JM for pre- and post-intervention focus groups. Each codebook was applied to raw data, and emerging themes, sub-themes, and categories were identified and summarized. Quotes were selected to highlight a typical statement made by study participants for each theme, representing both majority and minority perspectives.

### Ethical considerations

This study received ethical approval and oversight from the Kenya Medical Research Institute (protocol 2472) and the U.S. Centers for Disease Control and Prevention’s Institutional Review Board (protocol 6462). Both Institutional Review Boards authorized the investigators to obtain verbal informed consent from all participants. A waiver of documentation of consent had been requested since the research presented no more than minimal risk of harm to subjects, involved no procedures for which written consent is normally required outside of the research context, and to increase the comfort of rural study participants, many of whom were of low educational attainment and reluctant to sign forms they were unable to read. Personal identifiers of FGD participants were not recorded.

## Results

In total, 27 mothers and 25 nurses participated in the pre-intervention focus groups, and 42 mothers, 31 nurses, and 19 CHWs joined the post-intervention focus groups (Table 1). FGDs were held at healthcare facilities in the communities of Nyang’oma, Katito, Sondu, Masogo, Muhoroni, and Nyakach. Each focus group was well attended despite the late arrival of some participants.

Table 2 summarizes a list of themes, sub-themes, and categories identified in pre- and post-intervention FGDs for mothers, nurses, and CHWs. Analysis of FGD data revealed three major themes: 1) quality of care; 2) barriers to antenatal care and facility-based delivery; and 3) SWAP and OBA interventions. The sub-themes of *quality of care* include perceived quality of care at healthcare facilities, perceptions on home delivery, challenges with providing quality care, and factors that make nurses better providers. The sub-themes of *barriers to antenatal care and facility-based delivery* include perceived barriers to ANC visits and facility-based delivery and perspectives on mothers and community health workers. The sub-themes of *SWAP and OBA interventions* include proposed topics for text messages, overall experience with SWAP and OBA, experience with group education, and experience with text messages. To summarize the unique perceptions and experience of mothers, nurses, and community health workers and their changes from pre- to post-intervention FGDs, each sub-theme is discussed by the timing of FGD and the participant group below.

**Table 2:** Identified themes, sub-themes, and categories from pre- and post-intervention focus group discussions (FGDs) with mothers, nurses, and community health workers

Timing	Participant Group	Theme: SWAP and OBA Interventions	Theme: Quality of Care	Theme: Barriers to Antenatal Care and Facility-based Delivery
Pre	Mother	**Sub-theme: Proposed topics for text messages** -ANC appointment reminder -Family planning -Malaria -Water treatment -Nutrition -Immunization -HIV/AIDS	**Sub-theme: Perceived quality of care at health facilities** -Positive -Negative -Mixed **Sub-theme: Perceptions on home delivery** -Positive -Negative	**Sub-theme: Perceived barriers to ANC visits and facility-based delivery** -Money -Work -Transport -Safety and Security -Fear of mistreatment -Time -Supply shortage at healthcare facilities
	Nurse	**N/A**	**Sub-theme: Challenges with providing quality care** -Inadequate training -Limited equipment and supplies -Workforce shortage	**Sub-theme: Perceived barriers to ANC visits and facility-based delivery**-Poor roads -Stigma -Distance to healthcare facilities -Geographic terrains -Adverse weather
Post	Mother	**Sub-theme: Perceptions of group appointments** -Limited exposure -Beneficial to break cultural myths **Sub-theme: Perceptions of text messages** -Challenges with receiving messages -Language barriers **Sub-theme: Perceptions of intervention effects** -Positive -OBA more valued	**Sub-theme: Perceived quality of care at healthcare facilities** -Rude attitude and conduct of nurses -Sympathy for nurses **Sub-theme: Perceptions on home delivery** -Positive -Negative	**Sub-theme: Perceived barriers to ANC visits and facility-based delivery**-Fear -Lack of supplies and electricity -Cultural beliefs
	Nurse	**Sub-theme: Experience with SWAP and OBA interventions** -Positive -Unintended consequences -Remaining challenges **Sub-theme: Sustainability of existing programs** -Concerns for the termination of programs	**Sub-theme: Challenges with providing quality care** -Lack of equipment and supplies -Inadequate recognition -Limited teamwork -Inadequate compensation -Workforce shortage **Sub-theme: Factors that make nurses better** -Training -Specialization -Good supervision -Adequate staff and supplies -Rewards	**Sub-theme: Perspectives on mothers and community health workers** -Nurses’ attitude as a hindrance to ANC visits -Need for financial incentives for community health workers
	Community Health Worker	**Sub-theme: Experience with SWAP and OBA interventions** -Increased patients, ANC visits and hospital delivery -Challenges with product distribution availability -Benefits of health talks -Challenges with text messages **Sub-theme: Perceptions of training** -Satisfied -Not enough	**N/A**	**N/A**

### Pre-intervention FGDs: mothers

#### Proposed topics for text messages

The first major theme was SWAP and OBA interventions. In pre-intervention FGDs, the only intervention topic discussed was SWAP’s text messages. When FGD moderators asked mothers about health topics they would like to see addressed in text messages, participants expressed their interest in ANC appointment reminders, family planning, malaria, water treatment, nutrition, immunization, and HIV/AIDS. These responses informed text messages developed by SWAP.

#### Perceived quality of care at healthcare facilities

The second major theme was quality of care. At pre-intervention, mothers shared a variety of perceptions on the quality of care provided at ANC and delivery at healthcare facilities, ranging from positive to very negative. Positive perceptions of care included feeling welcomed and listened to by the nurses, and receiving a physical exam, medicines, and a clear, dated appointment for the next visit. Mothers also described a number of negative impressions of care that included being left to deliver on their own without any assistance and being slapped and verbally abused. One mother captured the uncertainty of the type of care they would receive, which could be determined by the provider assigned to them: “*Hospital delivery is good but only if you get a good nurse. Some of them really mistreat mothers, they insult you, and so it only depends on the nurse that you get”* (Mother, Pre-intervention FGD).

#### Perceptions on home delivery

The majority of mothers shared their recognition of home delivery with traditional birth attendants as an unsafe practice or at least a suboptimal practice. For example, mothers commonly noted that complications from pregnancy and delivery could be managed better at healthcare facilities than at home. Some mothers also believed that HIV transmission during delivery could be better prevented at healthcare facilities than at home and that injections were available for bleeding and post-pregnancy pain. Based on these positive perceptions of healthcare facilities, most of the mothers expressed that they wanted to deliver the next child at healthcare facilities despite the concern of mistreatment.

Conversely, home delivery was viewed as a favorable option by a few participants who had either experienced or heard about mistreatment of mothers by nurses. As one mother stated about home delivery: *“It is not safe but at least they don’t harass you the way the sisters [nurses] do”* (Mother, Pre-intervention FGD). Expressing a similar perspective regarding the kind of care given at home, other FGD participants said that local TBAs were “*serious*,” and were more likely to “*pamper*” and respond more quickly to the needs of laboring women than nurses in healthcare facilities. Some participants expressed their perspectives on the value of using traditional herbs to manage pain and facilitate delivery at home, believing that the herbs could help avoid surgery.

#### Perceived barriers to ANC visits and facility-based delivery

The third major theme that emerged from FGDs was barriers to antenatal care and facility-based delivery. Mothers listed a number of factors that inhibited women from visiting healthcare facilities for ANC visits and delivery, including lack of money, work, transportation, security and safety, fear of mistreatment by nurses, time, and shortage of supplies at healthcare facilities. Participants shared that people may not have money to pay for their first-time hospital fee of 100–150 Kenyan Shillings. For many women, work (e.g., drying maize at garden plots away from home) could prevent them from traveling to the hospital for ANC visits. More profoundly, one mother described how her concern about safety when traveling to the health facility at night would lead her to risk her health by staying home:

*“When labor starts in the morning, then I will come and deliver here at [the health center]. If it starts at night, it can be very risky. We live next to the road and when it reaches at night, there is a lot of chaos … I would not like to risk. So, if it is during the day, I will just come to the hospital, but if it is at night, then I will have to deliver at home*.” (Mother, Pre-intervention FGD)

Some mothers avoided ANC because many questions would be asked at the first ANC visit. The types of fear include HIV testing, injections, language barriers, and walking to the clinic while pregnant. A mother spoke about her fear of knowing her HIV status and explained that it delayed her timing of seeking care.

### Pre-intervention FGDs: nurses

Among nurses, two themes emerged from pre-intervention FGDs: challenges with providing quality care and perceived barriers to antenatal visits and healthcare facility deliveries.

#### Challenges with providing quality care

Nurses described several important challenges to providing quality care at healthcare facilities, including inadequate training, limited equipment and supplies, and workforce shortages. Nurses expressed the need for, and importance of, training and clinic updates to help them provide the most effective care and avoid outdated approaches to care. On resource limitations, one nurse stated:

*“Maybe you are in the labor ward, you have no delivery packs, and there is no water. Maybe there is somebody to be referred, and there is no vehicle. Maybe you are in the maternity, and you have no gloves. Those are some of the things that make my work difficult sometimes.”* (Nurse, Pre-intervention FGD)

Another nurse described an equally profound set of limitations in staffing:

*“I feel understaffing is the most challenging because if you are alone and you are pressed with a lot of work to do, you cannot really provide your services effectively. You are one person. You want to attend to the mothers. You are attending to the children. There you are giving injections. You are giving the drugs, and you are competing with time.”* (Nurse, Pre-intervention FGD)

#### Perceived barriers to antenatal care and facility-based delivery

Nurses identified a list of barriers to ANC visits and facility-based delivery that coincided with obstacles identified by mothers. These included poor roads, HIV stigma, distance to healthcare facilities, geographic terrain (e.g., steep hills), and adverse weather. One nurse stated that stigma associated with HIV/AIDS prevented women from returning to ANC visits or delivering at healthcare facilities once they found out their positive status.

### Post-intervention FGDs: mothers

Under the theme of SWAP and OBA interventions, maternal FGDs included the sub-themes of perceptions of group appointments (SWAP only), text messages (SWAP only), and intervention effects (both SWAP and OBA interventions).

#### Perceptions of group appointments

Organizing a system of group ANC appointments required SWAP to coordinate efforts with each healthcare facility, which delayed full implementation for several months. Consequently, FGD participants attended group appointments only once or not at all. Mothers who were able to attend group appointments commented on the benefits. For example, one mother noted that the appointments helped her overcome cultural myths:

*“Before I gave birth to my first-born, I used to hear people telling me that you are not supposed to buy clothes for the unborn baby. If you do that then, it is a taboo, and your child can die before it is born. So, I used not to buy clothes for the baby. When I came for those meetings, I was told that ‘those people are misleading you. Can you try and buy and then let us see if something will happen to the child?’ I bought the clothes, and there is nothing that happened to my baby. I also learnt how to take care of myself and the baby hygienically.”* (Mother, Post-intervention FGD)

Another mother shared that an additional benefit of the group appointments was the opportunity for social interactions:

*“I also knew one woman the first day that I attended the meeting. So, we left together, and as we were chatting on the way, we exchanged numbers, and we always communicate. Of late she called and asked me whether I still go back to the clinic, and I said yes.”* (Mother, Post-intervention FGD).

#### Perceptions of text messages

The text message intervention also had several challenges in the early stages. A number of mothers reported that they did not get ANC appointment reminders. One participant shared that while reading in Luo was difficult for her, she could speak the language well. Another said that when she first got a message, she thought it was sent to her by mistake, but then she saw SWAP’s name at the end of the message. FGDs also revealed that health education messages sometimes reached the phone of husbands instead of mothers:

*“I got my message through my husband’s phone. I was at home when the message arrived, and he was away from home. He told me that there was a message that he received from SWAP, but he accidentally deleted the message, so I did not get to read the message.”* (Mother, Post-intervention FGD)

#### Perceptions of intervention effects

Mothers shared their appreciation of both SWAP interventions and the OBA program:

*“They [SWAP interventions and OBA] are both important. One helped me because I gave birth free of charge, and the other one, every time I came from the clinic, I could fill my bag with the item that I was given at the shop there.”* (Mother, Post-intervention FGD)

Some participants valued the OBA program more than SWAP interventions because it substantially lowered economic barriers to care: *“They are both good, but I can say that OBA card was the best because it was catering for a lot of things that if we were to pay cash then maybe we wouldn’t”* (Mother, Post-intervention FGD).

#### Perceived quality of care at healthcare facilities

At follow-up, mothers continued to comment on nurses’ rude attitude, arrogance, and violent behaviors, However, perhaps in recognition of the increased workload engendered by the SWAP and OBA interventions, at least one mother shared her sympathy for the multiple tasks that burden nurses: “*There is only one nurse who is doing rounds in the wards, and she also wants to attend to you. If there could be at least more than one nurse, then things could be very easy”* (Mother, Post-intervention FGD).

#### Perceptions on home delivery

Some participants continued to express negative views on home delivery, recognizing that TBAs cannot respond to complications quickly and safely. After the concurrent implementation of SWAP interventions and OBA program, participants reported that home delivery can be costly:

*“What I can say about delivering at home is that it is not good because in the hospital, delivery is free unlike home. TBA wants to be paid. There is also some ashes that they give you when you feel labor to make the baby come out even when the time is not yet. This may make the baby die, and they end up taking your money as well”* (Mother, Post-intervention FGD).

#### Perceived barriers to ANC visits and home delivery

Barriers to care expressed in post-intervention FGDs were similar to pre-intervention FGDs, with the exception of economic barriers. Women still expressed their concerns about lack of supplies, fears of maltreatment and travel at night, and cultural issues.

### Post-intervention FGDs: nurses

#### Experience with SWAP and OBA interventions

At follow-up, nurses identified positive effects and remaining challenges of SWAP interventions and spoke to how SWAP interventions enhanced their work and service utilization. For example, nurses noted how group education was useful for organizing mothers: *“When I came to understand that we were going to book them as per the expected date of delivery, it really made our clinic to be organized”* (Nurse, Post-intervention FGD). Nurses also expressed appreciation for solar lighting that SWAP provided healthcare facilities that lacked electricity: “*… in the night when we have a blackout, we simply use the solar system. So, this has improved our uptake and the clients are able to deliver at night even when we have a blackout”* (Nurse, Post-intervention FGD).

Another benefit of the SWAP program noted by the nurses was the training received on neonatal resuscitation, obstetric emergency response, water and hygiene, and education through listening (a behavior change intervention on patient-centered care).

At the same time, FGDs uncovered some unintended consequences of SWAP interventions. A nurse shared that mothers come to deliver at healthcare facilities with an expectation that they receive incentives, so they did not come with necessary supplies for baby care. Another nurse noted that the increased workload had implications for care they delivered:

*“The negative part of it [increased clients] is that we have a high workload that sometimes cannot allow us to give quality service because we are struggling to finish the queue, and we miss giving some education to our clients. You find that when it comes to health talks like the danger signs, breast feeding education and other things, you find that time doesn’t allow us”* (Nurse, Post-intervention FGD).

Positive perceptions of the OBA program were also common among nurses. The program helped reduce nurses’ stress level during nights when they had difficult deliveries through increased availability of ambulance services to transport women to higher levels of care. Some nurses explained that the OBA program also provided additional financial resources for the healthcare facilities to conduct outreach activities and benefit people in remote villages.

The challenges with the OBA program described by nurses had less to do with the program itself and focused more on how the program magnified existing systemic problems, such as lack of equipment and supplies, and understaffing. By lowering economic barriers to care, OBA contributed to increased patient loads, which added to the stress of overworked nurses. Another challenge identified in FGDs was deficient transportation for referral: *For now, OBA is giving us support. They have paid for transport, but still the ambulance is the same one. You call. It’s not there"* (Nurse, Post-intervention FGD).

#### Program sustainability

Most of the participants expressed their hope to continue SWAP and OBA intervention activities and shared some concerns about the potential consequences of ending them. One nurse shared that new programs often *“come with support and staff, [and] upon exit [and removal of support staff], they integrate those services to the system … and leave it to nurses*” (Nurse, Post-intervention FGD). Nurses also discussed how CHWs may need financial incentives to continue providing antenatal education services after the program ends: *“You know even those people in the community they also have their priorities, the community workers. They have to provide for their families, and some other added duties”* (Nurse, Post-intervention FGD).

#### Challenges to providing quality of care

As noted above, although nurses’ perceptions of SWAP and OBA were generally positive, many had concerns about their ability to provide quality care because of shortages of equipment and supplies and understaffing. These resource constraints, along with inadequate recognition, teamwork, and compensation, were mentioned as factors that discourage nurses and lead to suboptimal care. Furthermore, FGD responses emphasized understaffing as the major challenge faced by nurses: *“It gives us a burnout, and even anger can flare out because expectation from outside is too much”* (Nurse, Post-intervention FGD). One nurse clearly summarized the consequences of these systemic problems:

*“At times, we also have attitude which sometimes really make these mothers not to come and deliver in the hospital. Many times you find that these mothers are saying that they will be slapped … . Sometimes the nurse will ask so many questions that they don’t like, and they are going to be intimidated. So, we should be well-staffed, and then as nurses, we should work on our attitude. Like how you talk to them in that situation will also attract the others to the hospital, and we provide the care”* (Nurse, Post-intervention FGD).

#### Factors that enable nurses to provide better care

Nurses also identified a number of factors that can enhance nurses’ productivity at work, such as training, specialization, good supervision, adequate staff and supplies, and rewards (e.g., financial rewards, acknowledgment of efforts). Several nurses noted that having proper equipment and better working conditions were *“most important”* for working effectively, and that *“there are places where nurses even work without gloves, without instruments, and even without the necessary essential drugs …*. I *think so many nurses are demotivated”* (Nurse, Post-intervention FGD).

### Community health workers

CHWs were included in post-intervention FGDs because they became important providers of care, moderating the group ANC appointments, providing educational talks, and handing out SWAP products as incentives. The sub-themes addressed in CHW FGDs centered on their experience with the SWAP and OBA programs, and their perceptions of the training they received.

#### Experience with SWAP and OBA interventions

CHWs discussed how SWAP and OBA interventions contributed to increasing ANC visits and hospital deliveries. One CHW noted that SWAP interventions made long waits worth their while: “*even if it was getting late, [mothers] could still persevere and wait because of the good thing that they were going to get*” (CHW, Post-intervention FGD). Another said that, before the SWAP program started *“the ANC turn up was very low but when we started giving mothers vouchers, the deliveries went up, and the number we were seeing in the ANC also went higher. They could come in large numbers, something that was not happening before”* (CHW, Post-intervention FGD).

The program was not without challenges because sometimes the incentives (health products) provided by SWAP ran out: *“You find that the products are brought around the fifteenth there [and run out after] only two weeks,”* so that women who had attended clinic after they ran out, *“never got or [had] seen any product”* (CHW, Post-intervention FGD).

CHWs reported that their group education sessions addressed nutrition, birth plans, malaria, and safe water practice (e.g., not carrying large amount of water). While a few challenges were identified, including late arrival of mothers and space limitation, the discussions uncovered how health talks benefited women and CHWs. Some CHWs paired up the mothers in the group sessions to share experiences, which helped even pregnant schoolgirls feel free to share their concerns.

CHWs also shared their experience with text messages, which included a similar challenge described by mothers in their FGDs:

*“The major challenge that I have encountered is that some young women come here. They give us their telephone numbers … and when they are called [by CHWs], you find that the husband quarrels claiming that who gave you my number, and so it is not easy to schedule a health talk since they say that the husbands will not agree”* (CHW, Post-intervention FGD).

#### Perceptions of training

CHWs discussed the training that they received prior to the SWAP intervention, and mentioned a list of topics, including record keeping, importance of hospital delivery, immunization, safe water, danger signs among pregnant women, nutrition, and basic business skills (e.g., reconciliation of product vouchers). A few CHWs shared that 2 days were not enough to understand record keeping practice and reconciliation, but many participants expressed their satisfaction with the training that they received from SWAP, and also the nurses:

*“We get to even ask the nurses about certain things that make us as well gain knowledge as they also get knowledge. This is then happiness to both of us”* (CHW, Post-intervention FGD).

**Fig. 1:**
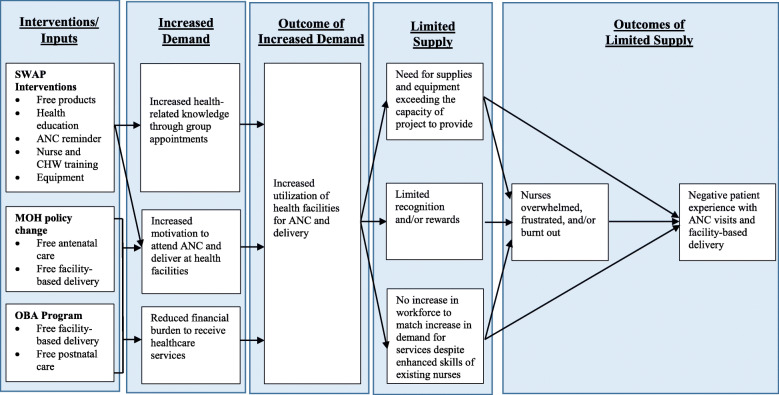
A conceptual model to explain how SWAP and OBA interventions increase demand for services, which increases service utilization, exceeding the supply of materials and personnel, resulting in the indicated outcomes related to quality of care, MSWANC Project, Kisumu County, Kenya, 2013–2014

Figure 1 illustrates a conceptual model to explain the impact of SWAP and OBA interventions on demand for, and supply of, ANC visits and facility-based delivery.

## Discussion

Baseline data in this evaluation highlighted the many challenges faced by programs that aspire to increase use, and improve quality, of antenatal and delivery services in public Kenyan healthcare facilities. Several important challenges for mothers included cost of care, distance from health facilities, lack of transport, fear of mistreatment by healthcare workers, safety (particularly during night-time hours), fear of HIV diagnosis, and supply shortages. Challenges faced by healthcare workers included inadequate training, insufficient equipment and supplies, lack of water in healthcare facilities, lack of emergency transport, and inadequate staffing with the attendant overwork and stress. Healthcare workers also noted barriers faced by mothers in seeking care, including bad roads, terrain, weather, distance to healthcare facilities, and the potential stigma of a new HIV diagnosis. These findings are consistent with other studies in different locations [9–13, 16, 19], and we summarized our findings in a conceptual model (Fig. 1).

The supply- and demand-side interventions implemented by SWAP intended to address some of the barriers described above. For example, previous studies have documented that demand-side incentives such as hygiene kits and insecticide treated bed nets contributed to increased antenatal service use among pregnant women by providing in-kind commodities valued by the women [12–14, 16, 17, 20, 21]. The findings of this study suggested that the free vouchers for commodities provided by SWAP may have motivated mothers to increase use of antenatal services. In particular, mothers acknowledged that they liked the vouchers, and healthcare workers and CHWs observed an increase in antenatal service use that they attributed to the vouchers.

Other demand-side interventions implemented by SWAP appeared to have beneficial effects on maternal perceptions of antenatal services. Mothers who participated in the group antenatal appointments said that the educational talks were useful and helped them overcome incorrect beliefs, and that they made new friends. Healthcare workers explained that the group appointments helped them organize care, and CHWs reported that they benefitted from the training, which helped them prepare mothers for their pregnancy. It was not clear from these interviews whether the group appointments increased ANC service use. Other research has suggested that group appointments can contribute to increased ANC service use in high-income countries [22, 23]. Because of its relatively low cost and high acceptability, this intervention merits further investigation of its utility as a motivator for ANC attendance.

Although the SMS appointment reminders did not reach many women, mothers did express a desire to receive them. Similarly, SMS educational messages had a modest impact for a variety of reasons, including lack of receipt by mothers, an inability by some women to read Luo (the local language), and husbands who did not want wives to share their mobile phone numbers. Nevertheless, some mothers who received them, and some husbands, valued the educational messages. Text messages have been successfully used as m-health interventions (e.g., use of mobile phones for health interventions) in developing countries [24]. More specifically, previous evaluations of programs to send SMS text messages to expectant mothers have shown promise in increasing service use [25, 26]. This approach deserves further study as a potential intervention to motivate increased use of care.

Supply-side interventions, which included training on emergency obstetric care, neonatal resuscitation, WASH, and patient centered care, were well-received by healthcare providers, but it was beyond the scope of this study to assess if these interventions increased demand for ANC services among pregnant women. Other studies have found that improved maternal health services have resulted in increased use [11, 27], but the interventions in this project may have been insufficient to motivate a measurable increase in participation by mothers. These interventions were likely insufficient to overcome the baseline perception by nurses that they had inadequate equipment and supplies. There is still a need to define the minimum package of providers, equipment, and supplies to provide optimal maternal care, and evaluate service use after implementation to assure they had the desired impact.

The OBA Card intervention, which was an incidental finding by our research team in intervention healthcare facilities, was a major confounder because of simultaneous implementation of SWAP vouchers and OBA Cards in the same healthcare facilities that made it impossible to tease out the specific effects of each intervention. Nevertheless, mothers appeared to prefer the OBA Card over the planned SWAP interventions because it lowered costs for obstetric care, permitted use of healthcare facilities that mothers perceived as providing better quality of care, and covered the costs of transporting women to healthcare facilities providing a higher level of care. These considerations were particularly important in western Kenya, where poverty limits mothers’ ability to pay for health services [2]. Comments by mothers in the FGDs suggested that, during the period of this project, the OBA Card and SWAP incentives may have complemented each other by motivating expectant mothers to attend ANC to receive vouchers for health products from SWAP and enabling them to obtain free deliveries in better quality healthcare facilities through the OBA Card. A separate review of the OBA Card program suggested that low-income populations benefitted through increased access to both public and private providers, [28] although another study found limited impact on use of postnatal care services [29].

While mothers in our FGDs appreciated SWAP and OBA Card programs and appeared to be motivated by them to increase service use, the programs also had unintended consequences, in particular, increases in healthcare provider workload in the context of insufficient workforce to meet increased demand for services and low pay. Similar challenges were faced in Kenya following institution of free maternal care in 2013, which resulted in increased use of antenatal and delivery services in public healthcare facilities by mothers [30], and increases in provider workload, inadequate staffing, and concomitant lack of motivation among healthcare workers in the face of inadequate funding and supplies [31]. Healthcare providers in this study also expressed concern about inadequate equipment and supplies, lack of resources, and little or no recognition of their effort. Nurses acknowledged that they were under pressure to manage multiple tasks at once, and that overworked health providers did abuse patients. These findings underscored the importance of increasing HCF staffing to meet increased patient demand for services in response to these interventions.

Despite the reported increase in service use motivated by the SWAP and OBA Card programs, follow-up data suggested that barriers to care persisted for some pregnant women. These barriers included fear of stigma if they were diagnosed with HIV, humiliating care, and abuse by nurses. Some mothers shared that they received inadequate care, or in some instances, did not receive proper attention for their delivery in healthcare facilities. The findings of mothers’ mixed experiences with facility-based care accord with previous studies in Ghana, Malawi, and Kenya that suggested that reprimands, social discrimination, and fear of chastisement by nurses at health facilities were factors that discouraged women from attending ANC [32]. Other research identified physical abuse, non-dignified care, discrimination, non-confidential care, abandonment of care, non-consented care, and detainment in facilities as important barriers to care experienced by pregnant women [33, 34]. In recognition of this problem, WHO has updated the Quality of Care Framework for Maternal and Newborn Health, highlighting the experience of patients as a key determinant of quality of care [35], and has developed public health interventions to improve mothers’ experience with antenatal care and delivery [36].

Although pregnant women detailed their negative experiences at healthcare facilities, some felt that health facilities were better able to manage complications and pain than traditional birth attendants. A review of qualitative research on facilitators and barriers to facility-based delivery from 17 countries suggested that some women regard healthcare facilities as the most respectable and safe locations for delivery [19].

This study had a number of limitations. First, researcher bias and reactivity among respondents potentially could have influenced the validity of study findings. Data collectors’ probes and reactions also may have altered how participants shared their perspectives. To minimize these validity threats, co-authors, including field staff, reviewed the findings to ensure that the major themes from FGDs were captured. Data collectors also received training on qualitative research and data collection to minimize their influence on study participants. Second, this study included few participants who received ANC reminder text messages. Consequently, we could not explore and highlight the experience of mothers who were exposed to all of the intended SWAP intervention activities. Third, because this evaluation was limited to women in one province of Kenya and used purposive sampling, findings may not be transferrable to other settings. Fourth, since recruitment for mothers occurred at healthcare facilities, results do not reflect the perspectives or experiences of mothers who exclusively used home delivery and did not pursue ANC during any point of their pregnancy. The percentage of women who do not seek antenatal care at healthcare facilities is less than 5%, but it is not known whether this group is at higher risk than health care users. Fifth, this study employed FGDs to collect qualitative data because Luo research assistants believed they were more culturally appropriate. We did not, therefore, conduct in-depth interviews, which may have been a more effective approach for eliciting personal experiences. Lastly, unbeknownst to SWAP staff at the time, the OBA Card intervention was introduced concurrently with SWAP intervention in intervention communities, creating confounding that could not be controlled for.

The limitations of this study were balanced by a number of strengths. Our team included two doctoral level behavioral scientists and two highly experienced, well-trained bilingual qualitative researchers who are native to the study region. Triangulation employed in the study design found consistent responses between study populations as well as consistency of themes identified independently by the investigators. The quotations used to illustrate themes reinforced the trustworthiness of the findings. In addition to two qualitative researchers who were native to the region, the other three team members had extensive experience in western Kenya as well as a deep understanding of the research topic. Finally, the analytic process is clear from the presentation of results.

## Conclusions

Based on in-depth qualitative data, this study explored the perspectives of mothers, nurses, and CHWs on their experience with SWAP and OBA Card interventions to promote ANC visits and facility-based delivery. Although demand-side interventions can increase service use by offsetting maternal costs of transport, time, and care, poor treatment by providers remains as a barrier to care. A more comprehensive intervention that includes additional workforce and adequate equipment and supplies are likely necessary to increase service use and respond adequately to increased demand for ANC services elicited by incentive programs. Additional research is needed to optimize packages of improved services and incentives to increase use of maternal and child health services, and to develop behavioral interventions to motivate nurses to treat patients with kindness and respect. In view of recent recommendations by WHO to increase the minimum number of ANC contact times to eight for reducing perinatal mortality [36], such interventions will be essential to motivate women to make the sacrifices necessary to increase the intensity of care.

## Supplementary information

**Additional file 1.** Supplementary File 1. Focus Group Discussion Guides. Baseline and follow-up focus group discussion guides for mothers and nurses, and follow-up focus group discussion guides for community health workers.

## Data Availability

The data used for the current study are available from the corresponding author on reasonable request.

## Abbreviations

AIDS: Acquired immune deficiency syndrome
ANC: Antenatal care
CDC: Centers for Disease Control and Prevention
CHW: Community heatlth worker
FANC: Focused antenatal care
FGD: Focus group discussion
HIV: Human immunodeficiency virus
OBA: Output Based Aid
PATH: US-based non-governmental organization
SMS: Short message service
SWAP: Safe Water and AIDS Project
USD: US dollar
UNICEF: United Nations Children’s Fund
WASH: Water, sanitation, and hygiene
WHO: World Health Organization

## References

[1] Alkema L, Chou D, Hogan D, Zhang S, Moller AB, Gemmill A (2016). Global, regional, and national levels and trends in maternal mortality between 1990 and 2015, with scenario-based projections to 2030: a systematic analysis by the UN maternal mortality estimation inter-agency group. Lancet.

[2] Kenya National Bureau of Statistics (2015). Kenya demographic and health survey 2014.

[3] Brown CA, Sohani SB, Khan K, Lilford R, Mukhwana W (2008). Antenatal care and perinatal outcomes in Kwale district, Kenya. BMC Pregnancy Childbirth.

[4] Bhutta ZA, Das JK, Bahl R, Lawn JE, Salam RA, Paul VK (2014). Can available interventions end preventable deaths in mothers, newborn babies, and stillbirths, and at what cost?. Lancet.

[5] Carroli G, Villar J, Piaggio G, Khan-Neelofur D, Gulmezoglu M, Mugford M (2001). WHO systematic review of randomized controlled trials of routine antenatal care. Lancet.

[6] World Health Organization (2002). WHO antenatal care randomized trial: manual for the implementation of the new model.

[7] Nair N, Tripathy P, Prost A, Costello A, Osrin D (2010). Improving newborn survival in low-income countries: community-based approaches and lessons from South Asia. PLoS Med.

[8] Manasyan A, Chomba E, McClure EM, Wright LL, Krzywanski S, Carlo WA (2011). Cost-effectiveness of essential newborn care training in urban first-level facilities. Pediatrics.

[9] Simkhada B, van Teijlingen ER, Porter M, Simkhada P (2008). Factors affecting the utilization of antenatal care in developing countries: systematic review of the literature. J Adv Nurs.

[10] Gabrysch S, Campbell OMR (2009). Still too far to walk: literature review of the determinants of delivery service use. BMC Pregnancy Childbirth.

[11] Brazier E, Andrzejewski C, Perkins ME, Themmen EM, Knight RJ, Bassane B (2009). Improving poor women’s access to maternity care: findings from a primary care intervention in Burkina Faso. Soc Sci Med.

[12] Kawakatsu Y, Sugishita T, Oruenjo K, Wakhule S, Kibosia K, Were E (2014). Determinants of health facility utilization for childbirth in rural western Kenya: cross-sectional study. BMC Pregnancy Childbirth.

[13] Mason L, Dellicour S, Kuile FT, Ouma P, Phillips-Howard P, Were F (2015). Barriers and facilitators to antenatal and delivery care in western Kenya: a qualitative study. BMC Pregnancy Childbirth.

[14] Fagerli K, O’Connor K, Kim S, Kelley M, Odhiambo A, Faith S, Otieno R, Nygren B, Kamb M, Quick R (2017). Impact of the integration of water treatment, hygiene, nutrition, and clean delivery interventions on maternal health service use. Am J Trop Med Hyg.

[15] Nanda P (2002). Gender dimensions of user fees: implications for women’s utilization of health care. Reprod Health Matters.

[16] Fleming E, Gaines J, O’Connor K, Ogutu J, Atieno N, Atieno S (2017). Can incentives reduce the barriers to use of antenatal care and delivery services in Kenya? Results of a qualitative inquiry. J Health Care Poor Underserved.

[17] Sheth AN, Russo ET, Menon M, Wannemuehler K, Weinger M, Kudzala AC (2010). Impact of the integration of water treatment and handwashing incentives with antenatal services on hygiene practices of pregnant women in Malawi. ASTMH.

[18] Creswell JW (2013). Qualitative inquiry and research design: choosing among five approaches.

[19] Bohren MA, Hunter EC, Munthe-Kaas HM, Souza JP, Vogel JP, Gulmezoglu AM (2014). Facilitators and barriers to facility-based delivery in low- and middle-income countries: a qualitative evidence synthesis. Reprod Health.

[20] Lindblade KA (2004). Sustainability of reductions in malaria transmission and infant mortality in western Kenya with use of insecticide-treated bednets: 4 to 6 years of follow-up. JAMA.

[21] Conrad P, De Allegri M, Moses A, Larsson EC, Neuhann F, Muller O, Sarker M (2012). Antenatal Care Services in Rural Uganda: Missed Opportunities for Good-Quality Care. Qual Health Res.

[22] Klima C, Norr K, Vonderheid S, Handler A (2009). Introduction of CenteringPregnancy in a public health clinic. J Midwifery Women’s Health.

[23] Patil CL, Klima CS, Steffen AD, Leshabari SC, Pauls H, Norr KF (2017). Implementation challenges and outcomes of a randomized controlled pilot study of a group prenatal care model in Malawi and Tanzania. Int J Gynecol Obstet.

[24] Gurman TA, Rubin SE, Roess AA (2012). Effectiveness of mHealth behavior change communication interventions in developing countries: a systematic review of literature. J Health Commun.

[25] Lund S, Nielsen BB, Hemed M, Boas IM, Said A, Said K (2014). Mobile phones improve antenatal care attendance in Zanzibar: a cluster randomized controlled trial. BMC Pregnancy Childbirth.

[26] Watterson JL, Walsh J, Madeka I (2015). Using mHealth to improve usage of antenatal care, postnatal care, and immunization: a systematic review of the literature. Biomed Res Int.

[27] Morris SS, Flores R, Olinto P, Medina JM (2004). Monetary incentives in primary health care and effects on use and coverage of preventive health care interventions in rural Honduras: cluster randomised trial. Lancet.

[28] Grainger C, Gorter A, Okal J, Bellows B (2014). Lessons from sexual and reproductive health voucher program design and function: a comprehensive review. Int J Equity Health.

[29] Warren CE, Abuya T, Kanya L, Obare F, Njuki R, Temmerman M (2015). A cross sectional comparison of postnatal care quality in facilities participating in a maternal health voucher program versus non-voucher facilities in Kenya. BMC Pregnancy Childbirth.

[30] Njuguna J, Kamau N, Muruka C (2017). Impact of free delivery policy on utilization of maternal health services in county referral hospitals in Kenya. BMC Health Serv Res.

[31] Wamalwa EW (2015). Implementation challenges of free maternity services policy in Kenya: the health workers’ perspective. Pan Afr Med J.

[32] Pell C, Menaca A, Were F, Afrah NA, Chatio S, Manda-Taylor L (2013). Factors affecting antenatal care attendance: results from qualitative studies in Ghana, Kenya and Malawi. Plos ONE.

[33] Bowser D, Hill K (2010). Exploring evidence for disrespect and abuse in facility-based childbirth.

[34] Bohren MA, Vogel JP, Hunter EC, Lutsiv O, Makh SK, Souza JP (2015). The mistreatment of women during childbirth in health facilities globally: a mixed-methods systematic review. PLoS Med.

[35] World Health Organization (2016). Standards for improving quality of maternal and newborn care in health facilities.

[36] World Health Organization (2016). WHO recommendations on antenatal care for a positive pregnancy experience.

