# Supplementary material for: The impact of supply-side and demand-side interventions on use of antenatal and maternal services in western Kenya: a qualitative study
Source: BMC Pregnancy Childbirth. 2020 Aug 8;20:453. doi: 10.1186/s12884-020-03130-4 (PMC7414717; doi:10.1186/s12884-020-03130-4)
Supplement: Supplementary file 1 — Additional file 1. Supplementary File 1. Focus Group Discussion Guides. Baseline and follow-up focus group discussion guides for mothers and nurses, and follow-up focus group discussion guides for community health workers. [file 12884_2020_3130_MOESM1_ESM.docx]

**S1: Focus Group Discussion Guides, baseline and follow-up, for nurses and mothers, and follow-up for CHWs**

**Focus Group Discussion Guide: Nurses**

*Attitudes, practices, and challenges regarding obstetrical care (baseline and follow-up)*

1. What aspect of your job do you like the most?
   1. Are you proud to be a nurse?
   2. Do you feel like you are good at your job?
      1. What could help you be a better nurse?
      2. What makes your job difficult?
   3. Do you feel like you are well-trained as a nurse?
   4. Are there ways you improve your skill? How?
2. How do you feel about providing obstetrical care?
   1. Do you feel like it is a good thing?
   2. Do you feel like it is a bad thing?
   3. Do you feel that it is necessary? Do you feel that it is important?
   4. What do you find most challenging about providing obstetrical care?
   5. What are some of the difficulties that you encounter on a daily basis while doing your job?
3. Do you feel like you are adequately equipped? *Probe for supplies and education*
   1. How many births do you usually do?
   2. How do you feel about that number? Would you like to do less? Would you like to do more?
4. If more women came to you for delivery, how would that affect your practice?
   1. How would that affect your health facility? *Probe for capacity, supplies*

*View about providing incentives to increase service use (follow-up only)*

1. How do you feel about giving mothers items like vouchers for health products or offering Ceramaji filters for purchase?
   1. Do you like it? Do you dislike it? Why?
   2. Do you receive any incentives for participating in these types of activities? If so, what?
   3. Your facility has been participating in an intervention where incentives, such as are provided to pregnant women attending clinic. How has this affected your practice? *Probe for time, capacity*.
   4. What were the benefits for the women?
   5. What were the challenges for you? What were the benefits for you?
   6. What do you think will happen if the incentives are discontinued?
2. What is your opinion about the OBA card program?
   1. What services has the program provided to women?
   2. Has this program changed your practice? How?
   3. Has this program changed the way women use services? How?
3. Did you receive additional training in this antenatal program?
   1. What are some of the skills that you were taught?
   2. Tell me about how the training was delivered. *Probe for: setting, instructor*
   3. Have you had the opportunity to use any of the skills that you were taught since then? If so, tell me about it.

*Feedback on the use of mobile phones for data transmission (follow-up only)*

1. Did the clinic you are working in receive a mobile phone to transmit data for this antenatal program?
   1. Were you trained in using the mobile phone? What are some of the skills that you were taught?
   2. Tell me about how the training was delivered. *Probe for: setting, instructor*
2. What has been your experience using the mobile phone?
   1. What were the challenges? What were the benefits?
   2. Do you like it? Do you dislike it? Why?

**Focus Group Discussion Guide: Mothers**

*Baseline*

*Water treatment behaviors*

1. How many people do you know of that treat their drinking water?
   1. Do they treat their drinking water every day? Most of the time? Some of the time?
   2. How often do you treat your drinking water?
   3. Where do you get your water treatment products?
   4. Do you think it is important to treat your water?
   5. Do think there is a connection between drinking water and illness?
   6. What are the difficulties in treating your water?

*Knowledge and attitudes about pregnancy and health care provided at antenatal, perinatal, and postnatal visits*

1. Would you share with us your experiences with your most recent labor?
   1. Where did you have your baby?
   2. What was it like for you to deliver your baby there? Do you think this was the best place for you to have your baby? Why?
   3. Did you have any complications with your pregnancy? If so, were there any signs early on that there were complications?
   4. Do you know of any signs during pregnancy or delivery that a mother or baby may be sick? How did you learn about these signs? *Probe for traditional birth attendants, clinic visits*
   5. Did you get a delivery kit?
      1. *If yes*, how did you find out about the delivery kit? What was in the delivery kit?
      2. How did the delivery kit affect your decision to deliver in the hospital?
      3. Did you pay for the delivery kit?
2. How do you feel about giving birth at home?
   1. Do you think it is safe?
      1. Is there someone in your family or village who could care for you if you got sick while you were in labor? Who?
      2. Is there someone in your family or village who could care for your newborn baby if they were sick? Who?
   2. What is nice about giving birth at home?
   3. What makes giving birth at home hard?
3. How do you feel about hospitals or clinics?
4. How do you feel about giving birth at a hospital or clinic?
   1. Do you know anyone who has delivered at a hospital?
   2. How many of your friends have delivered at a hospital?
   3. What would be an advantage of delivering at a hospital or clinic?
   4. What would be a disadvantage of delivering at a hospital or clinic?
   5. How would you get to the hospital?
   6. How long would it take you?
5. Where did you go for antenatal care?
   1. What was it like?
   2. How would you describe the nurse/doctor you saw?
   3. What kind of services did you get?
   4. Did you have to wait a long time?
   5. Did you receive anything at the visit?
   6. *If yes, w*hat did you receive?
   7. When were those items given to you?
   8. Did you hear about the items before you came into the clinic, or did you only find out about them when you were here?
   9. Were you given any education about the items you received?
   10. What did they tell you about them?
   11. Were the items only offered to you one time, or were they offered more than one time? When were they offered?
   12. Did you tell any of your friends or family about the items? What did you tell them?
   13. *For the items received:* What did you use them for?
   14. Would you recommend this to your friends or family? Why or why not?
6. Where do you think you will give birth (*probe for at home or at a hospital*)?
   1. Why?
   2. Do you think this is the best place for you to have your baby?
7. What is the biggest difficulty in attending clinic visits? *(Probe for transportation, time, other children, cost)* How do you get to the clinic or hospital?
8. How do most people in your village get to the hospital?
9. Is it difficult for you? What makes it difficult to get to the hospital? (*Probe for transportation issues, childcare issues)*
10. Is it expensive?
11. What would make it easier to get to the hospital?

*Evaluate the attitudes towards SMS text messaging as a health communication strategy for increasing the understanding of key safe motherhood messages*

1. If you were to receive text messages throughout this program regarding appointment reminders and tips for staying healthy during your pregnancy, do you think you would like that? Would that be helpful? What are some topics that might be most welcome to receive information about?
   1. What do you think about appointment reminders? And the health messages? Do you like them? Do you dislike them?

*Follow-up*

*Water treatment behaviors*

1. How many people do you know of that treat their drinking water?
   1. Do they treat their drinking water every day? Most of the time? Some of the time?
   2. How often do you treat your drinking water?
   3. Where do you get your water treatment products?
   4. Do you think it is important to treat your water?
   5. Do think there is a connection between drinking water and illness?
   6. What are the difficulties in treating your water?

*Knowledge and attitudes about pregnancy and health care provided at antenatal, perinatal, and postnatal visits*

1. Would you share with us your experiences with your recent labor?
   1. Where did you have your baby?
   2. What was it like for you to deliver your baby there? Do you think this was the best place for you to have your baby? Why?
   3. Did you have any complications with your pregnancy? If so, were there any signs early on that there were complications?
   4. Do you know of any signs during pregnancy or delivery that a mother or baby may be sick? How did you learn about these signs? *Probe for traditional birth attendants, clinic visits*
   5. Did you get a delivery kit?
      1. *If yes*, how did you find out about the delivery kit? What was in the delivery kit?
      2. How did the delivery kit affect your decision to deliver in the hospital?
      3. Did you pay for the delivery kit?
2. How do you feel about giving birth at home?
   1. Do you think it is safe?
      1. Is there someone in your family or village who could care for you if you got sick while you were in labor? Who?
      2. Is there someone in your family or village who could care for your newborn baby if they were sick? Who?
   2. What is nice about giving birth at home?
   3. What makes giving birth at home hard?
3. How do you feel about hospitals or clinics?
4. How do you feel about giving birth at a hospital or clinic?
   1. Do you know anyone who has delivered at a hospital?
   2. How many of your friends have delivered at a hospital?
   3. What would be an advantage of delivering at a hospital or clinic?
   4. What would be a disadvantage of delivering at a hospital or clinic?
   5. How would you get to the hospital?
   6. How long would it take you?
5. Where did you go for antenatal care?
   1. What was it like?
   2. How would you describe the nurse/doctor you saw?
   3. What kind of services did you get?
   4. Did you have to wait a long time?
   5. Did you receive anything at the visit?
   6. *If yes, w*hat did you receive?
   7. When were those items given to you?
   8. Did you hear about the items before you came into the clinic, or did you only find out about them when you were here?
   9. Were you given any education about the items you received?
   10. What did they tell you about them?
   11. Were the items only offered to you one time, or were they offered more than one time? When were they offered?
   12. Did you tell any of your friends or family about the items? What did you tell them?
   13. *For the items received:* What did you use them for?
   14. Would you recommend this to your friends or family? Why or why not?
6. Where did you think give birth (*probe for at home or at a hospital*)?
   1. Why?
   2. Do you think this was the best place for you to have your baby?
7. What was the biggest difficulty in attending clinic visits? *(Probe for transportation, time, other children, cost)* How do you get to the clinic or hospital?
   1. How do most people in your village get to the hospital?
   2. Is it difficult for you? What makes it difficult to get to the hospital? (*Probe for transportation issues, childcare issues)*
   3. Is it expensive?
   4. What would make it easier to get to the hospital?
8. Did you receive a free trial offer for a CeraMaji filter? When was it given to you? Did you hear about it before you came into the clinic, or did you only find out about it when you were here? Were you given any education about it? What did they tell you about it?
   1. Do you like it? Do you dislike it? Why?
   2. Did you purchase the filter through installment payments using M-PESA? Tell me about the process of paying installments. Was it easy? What were the challenges?
   3. What did you feel about the free trial? Did you like it? Did you dislike it?
   4. Would you purchase other health products through a similar process (i.e. free trial then installment payments?)
   5. Did you tell any of your friends or family about the filter? What did you tell them?
   6. Would you recommend this to your friends or family? Why or why not?

*Evaluate the attitudes towards SMS text messaging as a health communication strategy for increasing the understanding of key safe motherhood messages*

1. Did you receive text messages throughout this program regarding appointment reminders and tips for staying healthy during your pregnancy? Tell me about them? What did they say? Give me some examples if you can remember.
   1. What did you think about the appointment reminders? And the health messages? Did you like them? Did you dislike them?
   2. Did you tell any of your friends or family about the health messages you received? What did you tell them?

*Evaluate the attitudes towards ANC group meetings (Cohort meetings)*

1. Were you notified about group ANC meetings (health talks) during your pregnancy? How were you notified? What did you expect?
   1. Did you go to the meetings? How many group meetings did you attend? How many ANC appointments did you attend total? Were there many other women there? Did you know the other women? Did the same women attend multiple group meetings?
   2. What did you think about the group meetings?

*Evaluate the attitudes towards OBA cards and the potential positive and negative interactions with our vouchers/incentives*

1. How did you hear about the OBA card program? Did you join it?
2. What do you think about the OBA card program?
   1. How did you make use of the program?
   2. What impact did it have on your pregnancy?
   3. How did it compare with the voucher program?

**Focus Group Discussion Guide: CHPs**

*Follow-up*

1. How do you feel about giving mothers items like vouchers for health products for purchase?
   1. Do you like it or dislike it? Why?
   2. What were the challenges for you?
   3. What were the benefits for you?
   4. What were the benefits for the women?
   5. What do you think will happen if the vouchers are discontinued?
2. Did you receive additional training in this antenatal program?
   1. What are some of the skills that you were taught?
   2. Tell me about how the training was delivered. *Probe for: setting, instructor*
   3. Have you had the opportunity to use any of the skills that you were taught since then? If so, tell me about it.
3. What did you think about the health talks (group meetings)?
   1. What were the challenges for you?
   2. What were the benefits for you?
   3. What were the benefits for the women who participated?
   4. Would you like to keep doing this activity? Why or why not
